# If You Build It, Will They Come? Patterns of Internet-Based and Face-To-Face Participation in a Parenting Program for Military Families

**DOI:** 10.2196/jmir.4445

**Published:** 2016-06-22

**Authors:** Jennifer L Doty, Jessie H Rudi, Keri L M Pinna, Sheila K Hanson, Abigail H Gewirtz

**Affiliations:** ^1^ University of Minnesota Division of General Pediatrics & Adolescent Health Department of Pediatrics Minneapolis, MN United States; ^2^ Institute for Translational Research in Children's Mental Health Minneapolis, MN United States; ^3^ St. Catherine University Psychology Department St. Paul, MN United States; ^4^ University of North Dakota College of Business and Public Administration Grand Forks, ND United States; ^5^ University of Minnesota Family Social Science St. Paul, MN United States

**Keywords:** parenting, evidence-based practice, military, prevention, Internet, interactive media

## Abstract

**Background:**

Some evidence suggests parents are drawn to media-based interventions over face-to-face interventions, but little is known about the factors associated with parents’ use of Internet-based or Internet-enhanced programs, especially among military families. Research is needed to understand characteristics of parents who may be most likely to use online components or attend face-to-face meetings in order to ensure maximum engagement.

**Objective:**

In this study, we examined characteristics that predict various patterns of Internet use and face-to-face attendance in a parenting program designed for military families.

**Methods:**

An ecological framework guided analysis of differences in patterns of Internet-based use and face-to-face attendance by parents’ demographic characteristics (gender, education, employment, and child age), incentives offered, and number of months the parent was deployed. We reported differences in the total number of online components completed over the 14 modules, total number of face-to-face sessions attended, and the use of different types of online components accessed (videos, downloadable handouts, mindfulness exercises, knowledge checks, and downloadable summaries). Then, we computed multinomial logistic regression accounting for nestedness (parents within families) to examine associations between demographic, programmatic, and military-related characteristics and patterns of engagement (use of online components and attendance at face-to-face sessions).

**Results:**

Just over half (52.2%, 193/370) of the participants used the online components at least once, and the majority of participants (73.2%, 271/370) attended at least 1 face-to-face session. An examination of different patterns of participation revealed that compared with those who participated primarily in face-to-face sessions, parents who participated online but had little face-to-face participation were more likely to have received incentives than those who did not (95% CI 1.9-129.7). Among participants who had been deployed, those who had earned a 4-year degree (95% CI 1.0-2.2) and those who had been offered incentives to participate online (95% CI 2.1-58.6) were more likely to be highly engaged in online components and attend face-to-face compared with those who attended primarily face-to-face. However, those with a high number of months of deployment (95% CI 0.6-1.0) were less likely to be in the pattern of highly engaged in online components and face-to-face attendance. Compared with those who participated primarily face-to-face, deployed mothers were about 4 times more likely to engage in moderate online use with face-to-face attendance than deployed fathers (95% CI 1.21-11.83) and participate primarily online (95% CI 0.77-25.20).

**Conclusions:**

Results imply that parents may be drawn to different delivery options of a parenting program (online components vs face-to-face sessions) depending on their education level, incentives to engage in online components, and their military-related experience. Results suggest potential directions for tailoring Internet-based interventions.

## Introduction

Evidence-based parenting interventions have been shown to improve the well-being of both children and parents and have the potential to enhance family resilience in the face of chronic and acute stress [1-3]. However, the reach of these interventions is limited because of factors such as lack of trained practitioners and low parent participation [4-6]. These challenges have led researchers to begin examining parents’ preferences for online program delivery formats [7,8], which hold the promise for reaching far broader swaths of the population. Online program components (eg, videos that demonstrate key skills and downloadable handouts) are potentially an important supplement to traditional, face-to-face interventions [6,9]. However, we know little about which parents most engage with these program components.

More specifically, in military families, participation in interventions may be affected by the deployment of parents. A recent study revealed that more than half of active-duty military families who initially engaged in a prevention program did not complete it because of work-related issues or deployments [1]. Reserve component (ie, National Guard and Reserves; NGR) service members participate in monthly weekend drills and extended annual trainings in addition to their civilian work commitments. National Guard and Reserve populations also have been extensively deployed in the recent conflicts in Iraq and Afghanistan. There is a dearth of literature investigating the potential barriers to participation in parenting skills programs designed for military families, and particularly NGR families.

In this population, knowledge is also lacking regarding the relationship between demographic characteristics, ecological characteristics (eg, number of deployments), and parents’ use of online components in intervention settings. This information will allow service providers to tailor evidence-based programs for online distribution and maximize access to beneficial programs for military families. Online program components may be particularly beneficial to offset potential barriers to in-person participation. Understanding demographics and other characteristics of parents who engage in online components to differing degrees can inform resource allocation, recruitment, and retention efforts. To extend previous research on parent engagement in Internet-enhanced parenting programs, this study examined how demographic, programmatic, and military deployment-related factors are associated with patterns of parents’ use of online parenting components and face-to-face attendance in a parenting program for military parents.

### Engaging Parents Online

#### Online Parenting Programs

A growing number of Internet-based interventions have been geared specifically toward parents and parenting [10,11]. One study examined the efficacy of an 8-module, intensive, positive parenting program, Triple P Online, for parents of children with early-onset disruptive behavior problems using a randomized controlled trial (RCT) design [12]. Parents receiving the Internet-based intervention had significantly better outcomes on measures of children’s problem behavior, dysfunctional parenting styles, parents’ confidence in their parenting role, and parental anger compared with the control group (general Internet use). These gains were generally maintained, and in some cases enhanced, at the 6-month follow-up assessment. Additionally, satisfaction ratings for the Internet-based intervention program were high [12].

Similarly, another RCT evaluated the efficacy of an Internet-based parent management training program for children with conduct problems [13]. Children whose parents had participated in the Internet-based program showed a greater reduction in conduct problems than children in the waitlist control group. Parents who completed the Internet-based parenting program also reported less use of harsh and inconsistent discipline after completing the program, as well as more positive praise. These positive effects were maintained at the 6-month follow-up. Other RCTs have also found that Internet-based parent management training programs are effective in reaching parents and report encouraging results [10,14-17].

Although these randomized trials are important for establishing the efficacy of Internet-based programs, they revealed very little about which parents might gravitate toward a face-to-face program and which might gravitate toward online materials because all intervention families were assigned to the Internet-based treatment. Often, Internet-based programs are viewed as a way to reduce barriers to engagement, but research comparing in-person versus Internet-based programs (ie, comparative effectiveness trials) is lacking. Studies that include Internet-based programming as a supplement to a face-to-face program can begin to answer some of these questions about differences in parents’ online usage. Sanders and colleagues [18] found that online supplemental materials were as effective as self-help workbooks for parents. In addition, Internet-based supplements to a parenting prevention program presented via television were found to be an effective method of engaging hard-to-reach individuals [19]. Little is known, however, about what contextual factors—individual, familial, or ecological—are associated with parents’ use of Internet-based programs and supplements compared with face-to-face programming.

#### Parents’ Preferences

Efforts to understand how parents engage in parenting programs have used marketing methods to explore preferences for program delivery options [7]. Cunningham and colleagues [7] identified subgroups of parents who preferred different resource delivery formats. The “Action” segment of parents (ie, those highly motivated to engage in services) preferred weekly, in-person meetings with other parents in addition to coaching phone calls from a therapist, whereas the “Information” segment of parents preferred to solely receive parenting information without in-person meetings or support [7]. The “Overwhelmed” segment of parents was more likely to have a child with externalizing problems but was less likely to prefer information or professional support than other segments of parents. Interestingly, however, overwhelmed parents reported preferring information found on the Internet significantly more than those in the other two segments. Metzler and colleagues [8] found a preference among parents for media-based programs (eg, television, online) rather than face-to-face programs in a survey of 162 parents with children aged 3-6 years. Overall, parents reported preferring television programs, online formats, and written materials to face-to-face interventions. Program materials that are tailored to parents’ individual preferences may improve engagement in and adherence to Internet-based parenting programs [7,20].

The studies discussed above examined parent preferences, rather than actual behavior. Moreover, some evidence suggests that parents’ preferences do not always match actual engagement in various methods of program delivery, and more information about parents’ behavior in practice is needed [7]. This study fills a gap in the literature by examining the use of Internet-based programming and patterns of face-to-face attendance in a parenting program for military families and the characteristics and contexts that predict those patterns.

### Demographic Characteristics Associated With Online Use

Although little is known regarding parent characteristics related to adherence to Internet-based prevention programs, parent and family demographic variables have been shown to be related to parents’ general use of the Internet and other technology. Specifically, income and education were associated with parents’ online information-seeking behaviors [21-23]. In another study, parents who were on a waitlist for mental health services for their children and preferred Internet support had higher levels of educational attainment compared with those who preferred group or face-to-face interim services [24]. Although seeking information online differs from participating in an Internet-based parenting program, these studies help inform our understanding of parents’ use of Internet-based programs.

Gender is another important demographic characteristic to consider. Although some research has found that women do not use the Internet as frequently as men [25], other studies have found no gender differences in Internet use [26]. However, mothers have tended to use more parenting content online than fathers [27,28]. A study of parents’ preferences for online, parenting video episodes found that mothers tended to rate the online modules as more engaging, watchable, and realistic than fathers did [8]. Mothers may find parenting content delivered via media, such as television and the Internet, more interesting and relevant compared with fathers. Existing parenting content may be tailored more to mothers’ unique needs than fathers’ [29]. Alternatively, mothers may be gatekeepers to fathers’ access to parenting content [30] or fathers may lack parenting efficacy given the traditional role mothers have played in child-rearing [31]. Together, the aforementioned studies suggest that parents’ demographic characteristics are linked to online preferences and use.

### Face-To-Face Attendance

The greater body of research on engaging parents in face-to-face parenting programs may inform research on Internet-based program participation. Face-to-face attendance in parenting programs varies widely; studies report that parents attend 35% to 61% of face-to-face group sessions [4,32,33]. A meta-analysis found a small but significant negative effect of socioeconomic status (SES) on dropout rates [34], addressing prior research that had reported mixed findings in this regard [35-37]. Similarly, single parents have been found to attend fewer face-to-face sessions than married or cohabiting parents in some studies [38] but not in others [32,39]. Family size is also related to parents’ face-to-face attendance in parenting programs, such that those with large families tend to attend fewer face-to-face sessions [32]. Overall, the evidence suggests that families at slightly higher risk (ie, single-parent families, lower SES families, and those with more children), who arguably may benefit most from such programs, may actually attend fewer sessions and participate less [32,38,40].

Evidence also suggests that process variables within the prevention program experience (eg, interest in and comfort with the group) may be related to continued participation [40]. For example, Fox and Gottfredson [41] found that parents who completed the program tended to report higher interest and more comfort with the pretest or program participation than parents who did not complete the program. In another study, interaction with other participants and group dynamics contributed to retention [40].

Although parents report that small incentives would promote attendance [42], studies have had mixed results regarding the effectiveness of providing incentives to encourage attendance. Al-Halabí Díaz and Errasti Pérez[43] found that the use of small incentives at the end of each session improved face-to-face attendance. Similarly, other studies found that incentives were positively associated with face-to-face attendance [44,45]. However, a relationship between incentives and attendance has not been found in other studies [46,47]. Although much has been written about attendance in face-to-face parenting programs, much less is known about participation in online settings.

### Ecological Characteristics

From an ecological systems framework, families’ ability to participate in parenting programs may be influenced by barriers or facilitators in their immediate environment [48,49]. On the basis of the ecological assumption that basic resources in the environment sustain families and communities [48], those who most need support may also be those who lack the resources to access that support. However, while the aforementioned literature outlines barriers for other at-risk populations, very little is known about the barriers to participating in parenting programs for military families.

Among active duty military families, levels of practical and emotional support may be high because of a sense of community found on military bases [50]. However, NGR families experience more isolation, as they are geographically dispersed across the United States without access to resources found on military installations. National Guard and Reserve parents may also feel especially pressed for time as many have civil employment during the week and military training and duties on weekends [1], and this may be a substantial barrier to face-to-face participation in parenting programs.

Internet-based resources may provide support to parents who feel isolated as a result of a partner’s deployment. Although technology has become increasingly accessible [51], little is known about which members of military families are most likely to engage with Internet-based resources. Evidence suggests that the deployment cycle in particular may increase families’ use of technology to stay in touch [52,53]. During deployment, parents who are on the home front, the majority of whom are mothers, are accustomed to the use of Internet-based technology for communication with deployed service members. Research also suggests Internet-based programming may be well suited for military families post deployment [54]. However, those who have extensive prior deployments may be less likely to engage in parenting programming if they are struggling with multiple demands to reenter civilian life or posttraumatic stress symptoms [55].

### Our Study—After Deployment, Adaptive Parenting Tools

In our study, we sought to identify patterns of Internet-based and face-to-face participation in After Deployment, Adaptive Parenting Tools (ADAPT), an Internet-enhanced parenting program for military families. We examined the demographic, programmatic, and military-related characteristics associated with different patterns of engagement. Understanding patterns of engagement is important because the success of prevention programs and the benefits parents receive from these programs depend on their ability to engage and retain parents. This study analyzed data from an RCT of ADAPT, which aimed to improve parenting and child adjustment in families after the reintegration of a parent from deployment to Iraq or Afghanistan. The ADAPT program is a 14-week parent training program that provided online components as supplements to weekly face-to-face programming.

To understand patterns of engagement with online components and face-to-face participation with military parents, we sought to answer the following research question: How often did parents use various online components and attend face-to-face sessions? Furthermore, based on the ecological systems framework, we hypothesized the following:

- H_1_: Characteristics of parents (higher income, more education, full or part-time employment, fewer children, younger children, married, and female) and the program (incentives) will be positively associated with patterns of greater online and face-to-face use.
- H_2_: Characteristics that may pose a barrier to military families’ participation (number of deployments) will be positively associated with patterns of lesser online and face-to-face use.

## Methods

### Procedures

This study includes the subset of families from the larger study (N=336 families) who were randomly assigned to the treatment group—that is, to participate in a preventive intervention program, ADAPT (n=207 families; n=370 parents). Families were eligible to participate in the RCT if at least one parent had been deployed overseas in service of Operation Enduring Freedom (Afghanistan), Operation Iraqi Freedom (Iraq), or Operation New Dawn (Iraq) since 2001, if the family had at least one child between the ages of 4 and 12 years, and if they were willing to participate in the parenting program if invited. Participants were recruited in several ways: project staff presence and presentations at military-sponsored events (eg, reintegration events and military family picnics), referral from military personnel (eg, family readiness group leader or commander), word of mouth from fellow service members or another parent, and media (eg, television or radio, advertisements, and online social media). Families could go directly to the ADAPT website to consent to participate or request to be contacted for more information. Recruitment staff replied via phone call to answer questions, emailing the hyperlink for the screener and online consent form as needed.

In two-parent families, both parents were invited to participate in the online and in-home baseline assessments after individually completing the online consent form. After baseline assessments, families were randomized to either the ADAPT parenting program or the control group (services-as-usual). Staff then contacted the family to inform the parents of the result of randomization and discuss arrangements for those who were randomized to ADAPT to attend group sessions. Specifically, groups were arranged to be delivered in parents’ geographic area, as close to their home as possible. If no groups were currently occurring in their geographic area, parents were invited to the next available group after their baseline assessment. As these arrangements were made for determining which geographic group a family would attend, staff also encouraged both parents in two-parent families to attend the program. Staff explained to such families that although attendance by both parents was expected to be most beneficial, it was not required (eg, if one parent had to work, the other parent could attend alone). A total of 7 cohorts of groups were delivered, each cohort included 2 to 7 groups, and each group included 3 to 10 families (up to 16 individual parents per group).

### After Deployment, Adaptive Parenting Tools Program

The ADAPT program is an adaptation of Parenting Through Change, a 14-week group-based Parent Management Training–Oregon Model (PMTO) preventive intervention, for military families [56]. In addition to targeting positive parenting practices that are core to PMTO interventions (skill encouragement, positive involvement, family problem solving, monitoring, and effective discipline), ADAPT provides intensive coaching for parents on emotion regulation (via mindfulness exercises) and emotion coaching skills [57]. Skills are taught in 14 two-hour weekly group sessions using active teaching methods such as role-playing and group discussions. For more information about the ADAPT program, see Gewirtz and colleagues [54,57].

#### Online ADAPT Components

Building upon previous research demonstrating the many barriers to face-to-face attendance at prevention programs [5] as well as difficulties with retention [37,44], an Internet-based supplement was developed to engage parents with the program content in their own homes as much as possible (see Multimedia Appendix 1 for example screenshots). The 56 online components were supplemental materials organized by the 14 group sessions into 14 online modules. Each module included a menu of components such as access to skill and practice videos of military families who are learning and practicing key parenting skills with their children, audio recordings of mindfulness exercises, knowledge checks, and printable PDF documents summarizing key parenting skills. Parents were able to choose the components that best fit their needs for a personalized approach to the online materials. The number of components in the modules ranged from one in module 14 to eight in module 5. After each group session, parents received an email prompt directing them to the relevant online module for that week. ADAPT facilitators encouraged parents to view online material between sessions and to share the material with family members. Parents who were unable to attend face-to-face sessions were given the option to complete the online program modules when delivery of group sessions was complete (ie, after all possible face-to-face attendance options had been exhausted).

#### Online and Face-To-Face Program Incentives

In an effort to encourage online engagement after initial low participation online, those in cohorts 3 through 7 who used the online components the previous week were entered into a drawing for a US $25 gift card at the face-to-face group session. Families participating in face-to-face sessions received a US $15 gift card for attendance at each session to compensate them for their travel and time. Participants who did not attend face-to-face sessions but were offered to complete the program online in the last cohort were provided a US $15 gift card at the completion of each online session.

### Measures

#### Use of Online Components

Parents’ use of the online components was recorded using an online data tracking system. The system recorded whether each parent accessed an online component via clicking (coded as 0=did not click, 1=click). Clicks were interpreted as parents accessing and using that online component, and date stamps showed that parents accessed modules consecutively on different days, an indication that they were not casually browsing the material at one sitting. The total number of all components used was calculated as well as the total number of each component type used (videos, mindfulness exercises, knowledge checks, and downloadable summaries). In an effort to maximize use of online components, incentives were provided to families in cohorts 3 through 7 and in the online cohort (0= *did not receive incentives to go online*; 1= *received incentives to go online*).

#### Face-To-Face Session Attendance

Participants’ attendance at face-to-face sessions was recorded using sign-in sheets, receipts from gift card payments, and facilitator records. Number of sessions attended was summed to create a variable indicating the number of face-to-face sessions attended (of those who attended at least one session: mean 8.50, SD 3.91; including those who did not attend: mean 6.26, SD 5.04).

#### Variables Relevant to Military Families

Parents who had served in the military reported the number of months they had been deployed in the recent conflicts since 2001. A dichotomous variable reflecting deployment was created for the entire sample (0= *not deployed*; 1= *deployed in recent conflicts*).

#### Demographics

Parents self-reported demographic characteristics at their initial in-home assessment. Socioeconomic variables included household income in US dollars (less than $10,000 per year; $10,000 or more per year in $10,000 increments up to $150,000; or more than $150,000 per year) and education level (some high school or less, General Educational Development test, high school diploma, some college, associate’s degree, 4-year college degree, master’s degree, or doctoral or professional degree). Parents reported the number of children living at home at least 50 percent of the time, target child’s date of birth (converted to age), marital status (dichotomized into married and not married), and employment status (dichotomized into employed full-time or part-time and not employed).

### Data Analysis

We addressed the research question regarding the frequency of parents’ online component use and face-to-face attendance using descriptive analyses (*t* tests and correlations). We also examined the use of different types of online components accessed, including videos, mindfulness exercises, knowledge checks, and summaries using descriptive analyses. We then identified patterns of use of online components and face-to-face attendance, and parents were categorized into descriptive groups based on their frequency of participation online and face-to-face. To test the hypotheses that demographic, programmatic, and military-related variables would predict patterns of use, multinomial logistic regression with standard errors adjusted to account for the nested data structure (ie, parents within families) was computed in Stata 14 [58]. We examined associations between demographic characteristics (education, income, employment, number of children, marital status, child age, and gender), cohort (incentive to participate online), and military-related characteristics (months deployed) and patterns of online and face-to-face participation. Because deployment and gender were confounded (almost all men were deployed), additional analyses included only those who had been deployed.

Missing data analysis revealed very little missing data. Only 2 variables had missing data that made up more than one percent of the total sample: number of children (1.9%) and income (1.4%). Therefore, listwise deletion was used, resulting in an analytic sample of 365 individuals in the main multinomial logistic regression model.

## Results

### Descriptive Statistics

Of the 207 invited families, 84.5% (175/207) participated in at least one of either the online modules or the face-to-face sessions. A total of 75.4% (156/207) participated face-to-face and a total of 68.6% (142/207) participated online (9.2%, 19/207, participated only online). Of the participating individuals, 88.3% (325/368) were married, and on average, most parents reported having 2 or more children. Parents were asked to choose their youngest child between the ages of 4 and 12 years to be the primary focus child for the assessments. The average age of families’ target child was 7.7 years. Parents reported annual household income as follows: 25.5% (93/365) had an annual income of less than US $50,000; 43.3% (158/365) had an annual income between US $50,000 and US $99,999; and 31.2% (114/365)had an income of more than US $100,000.

We also analyzed parents’ engagement in the program as individuals: 78.9% (292/370) participated in either the online modules or the face-to-face sessions. A total of 73.2% (271/370) participated face-to-face and a total of 52.2% (193/370) participated online (5.7% participated only online; 21/370). Approximately half of the participants were mothers (51.1%, 188/368), and 90.5% reported being European American. Parents reported belonging to the following branches of the military: Army National Guard or Reserve (44.3%, 163/368), Air Force National Guard or Reserve (7.9%, 29/368), Navy Reserve (1.6%, 6/368), and other (6.8%, 25/368); 39.4% (145/368) were civilian partners. On average, the number of deployments for service members was 1.7, and the average number of months of deployment was between 13 and 18 months.Of the participants, 50.3% (185/368) had completed a 4-year degree or more and 67.1% (247/368) were employed full-time.

### Online and Face-To-Face Participation

**Figure 1:**
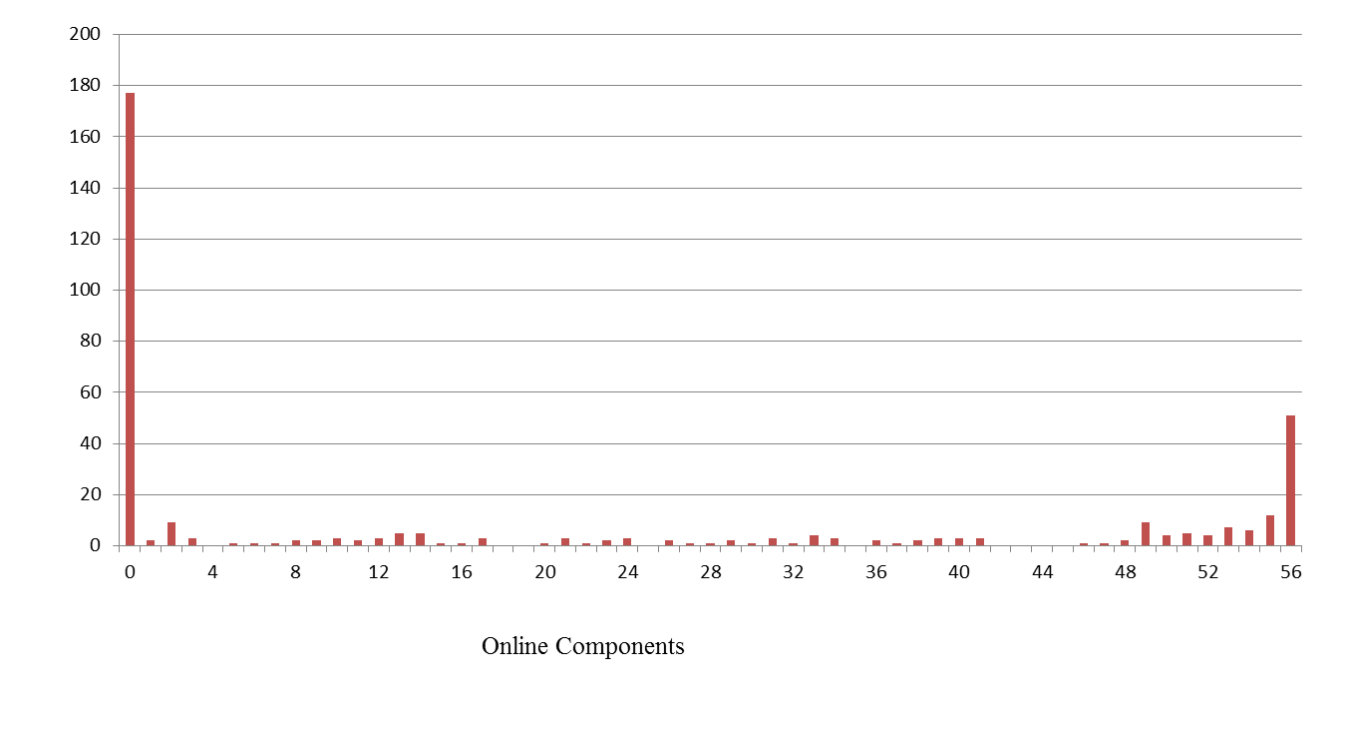
Histogram of Online Components Participants Accessed (n=370).

**Figure 2:**
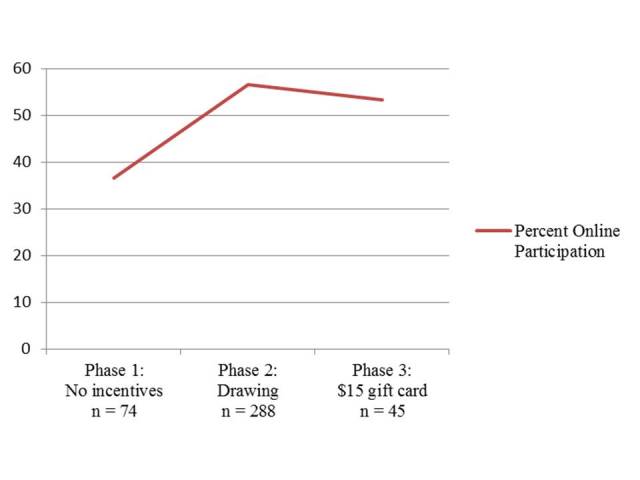
Graph of 3 phases of incentives for online participation (n=370).

Descriptive analyses revealed that the overall pattern of use of online components was bimodal (ie, no use or high use; Figure 1). The percentages of mothers and fathers who accessed each component at least once were calculated (Table 1). Because the distribution was bimodal (as has been found in past trials of PMTO, those who participated at least 3 times were more likely to complete the program), a cutoff of 4 components was used to calculate both online and face-to-face use. Among participants who accessed at least 4 online components, the mean number of components accessed was 41. Online videos and handouts were accessed most frequently. The first components in the first modules had the heaviest use. Significant differences in online component use were found by gender, education, incentives, and past deployment (Table 2). Mothers, parents with a 4-year degree, parents who received incentives to participate online, and those who had not been deployed used more components compared with fathers, parents without a 4-year degree, parents who did not receive incentives to go online, and those who had been deployed. In Figure 2, a graph illustrates the rate of online participation in 3 phases of incentives to go online: phase 1, no incentives; phase 2, a drawing for those who went online; and phase 3, gift cards for those who went online. Correlations revealed a significant but trivial, positive relationship between online use and number of months deployed (.10, *P*=.050).

**Table 1:** Parents’ use of online components (n=370).

Component	Total parents		Mothers		Fathers	
	n	%	n	%	n	%
Any online use	193	52.2	115	60.5	78	43.3
Videos	185	50.0	113	59.5	72	40.0
Knowledge checks	170	45.9	102	53.7	68	37.8
Mindfulness	176	47.6	109	57.4	67	37.2
Handouts	183	49.6	110	57.9	73	40.6

**Figure 3:**
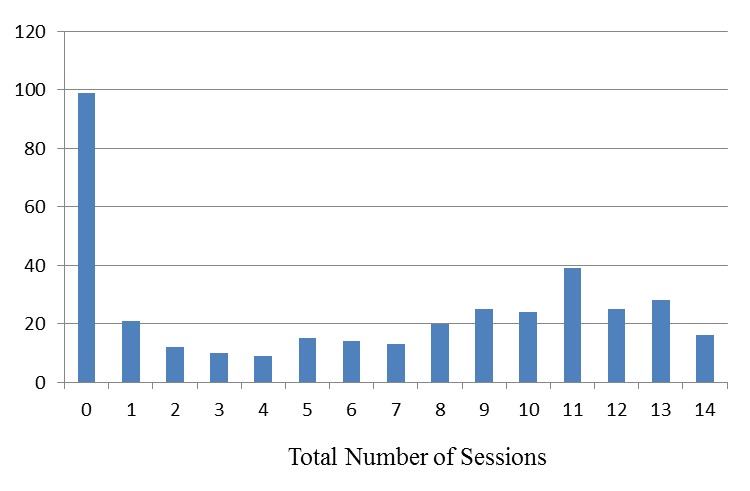
Histogram of Total Number of Face-to-Face Sessions Participants Attended (n=370).

Face-to-face attendance was also bimodal (Figure 3). Among participants who attended at least 4 sessions, the average number of sessions attended was 9.8. Mothers attended marginally more than fathers (Table 2). Correlations revealed a statistically significant, negative relationship between face-to-face attendance and child age—that is, parents with younger children attended more sessions (−.14, *P*=.009).

**Table 2:** Results of *t* tests of participation in online components and attendance.

Online Components	Gender			Education			Incentives			Deployed		
	M^a^	F^a^	*P*	<4 years	4 years+	*P*	Y^a^	N^a^	*P*	Y	N	*P*
Total	17.0	23.2	.013	18.1	22.2	.104	23.0	8.4	<.001	18.2	22.7	.077
Videos	4.9	6.5	.007	5.2	6.2	.109	5.1	2.2	<.001	5.1	6.6	.023
Knowledge checks	1.8	2.6	.008	2.0	2.4	.088	2.5	0.9	<.001	2.0	2.5	.060
Handouts	3.7	4.7	.055	3.8	4.7	.095	4.9	1.8	<.001	3.9	4.7	.166
Mindfulness	3.9	5.4	.011	4.2	5.1	.155	5.4	1.4	<.001	4.2	5.3	.063
Attendance	5.8	6.7	.088	6.4	6.1	.560	—	—	—	6.08	6.49	.444

^a^ M: male; F: female; Y: yes; N: no.

**Figure 4:**
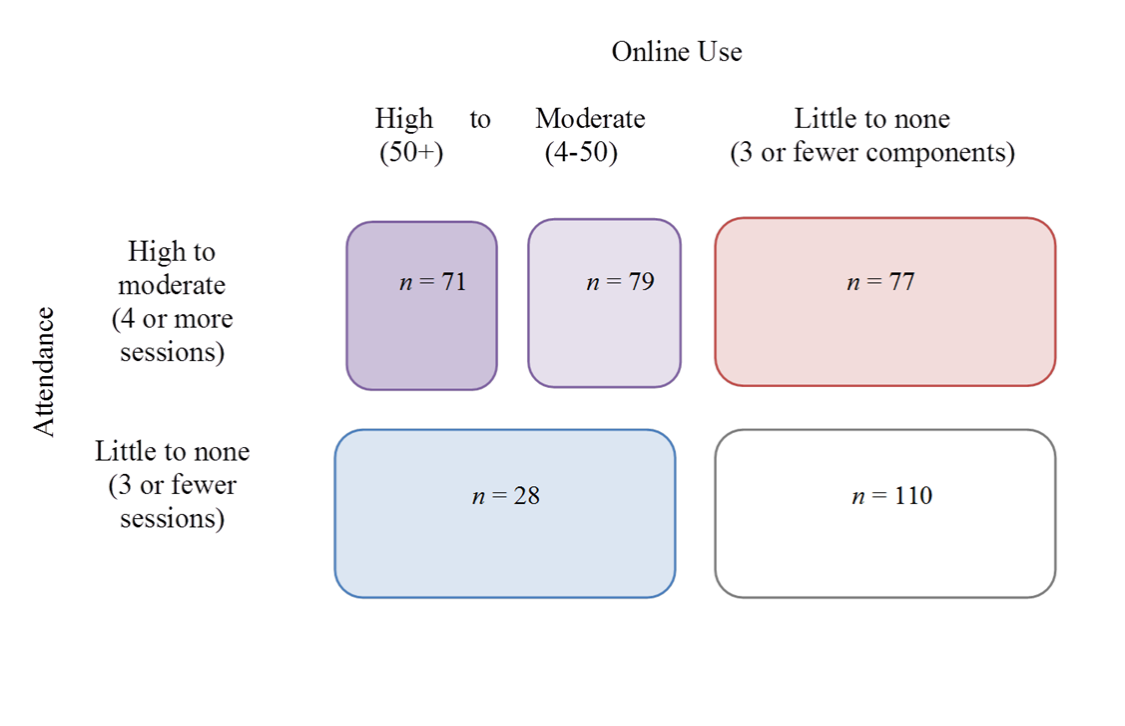
Patterns of online use and face-to-face attendance (analytic sample; n=365).

Guided by the distributions of online component use and face-to-face attendance (Figures 1 and 3), five patterns of program engagement were identified (Figure 4): (1) face-to-face attendance with high online use, (2) face-to-face attendance with moderate online use, (3) primarily face-to-face attendance, (4) primarily online use with little face-to-face attendance, and (5) little to no attendance or online use. The difference between high and moderate online use was determined by a median split of the number of online components used.

To test our hypotheses that demographic, programmatic, and ecological characteristics (ie, military related) would be associated with various patterns of participation, multinomial logistic regressions were computed, adjusting the standard error to account for individuals nested in families. The reference group of parents who attended primarily face-to-face was chosen because it represented traditional delivery. Because the vast majority of the men in this sample had been deployed, gender and deployment were confounded and modeled separately in preliminary models. The first hypothesis that demographic and programmatic characteristics would be associated with patterns of greater online and face-to-face use was partially supported. The following findings note significant differences with a *P* value of less than .05 compared with the reference group, parents who attended primarily face-to-face. The first model fit the data well (χ^2^_20_=72.8, *P*<.000), and gender was a significant predictor of each pattern (results available upon request). The second model, in which we removed gender but added deployment, also fit the data well (χ^2^_20_=62.9, *P*<.000), and deployment was a significant predictor of each pattern (see Tables 3 and 4). Those who had face-to-face attendance and high engagement online had higher levels of education. Those who were employed and who had older children were more likely to have a pattern of little face-to-face or online participation. Those who were incentivized to go online were about 2 to 9 times more likely to have a pattern of face-to-face attendance and high or moderate online use and about 15 times more likely to have a pattern of primarily online use. Income, number of children, and marital status were not associated with patterns and were therefore removed from the models for parsimony.

**Table 3:** Multinomial logistic regression of characteristics associated with patterns of online component use and face-to-face attendance (n=365).

Characteristic	Attendance + high online use (n=71)			Attendance + moderate online use (n=79)		
	RR^a^	*P*	95% CI	RR	*P*	95% CI
Deployed	0.44	.017	0.23-0.87	0.47	.027	0.24-0.92
Education	1.38	.025	1.04-1.84	1.21	.162	0.93-1.59
Employed	0.75	.518	0.32-1.79	2.17	.099	0.86-5.45
Child age	1.13	.094	0.98-1.29	1.10	.150	0.97-1.25
Incentives	9.69	.000	3.23-29.03	1.96	.086	0.91-4.23

^a^RR denotes relative risk. Reference group attended at least 4 times but had little to no online use (n=77).

**Table 4:** Multinomial logistic regression of characteristics associated with patterns of online component use and little face-to-face attendance (n=365).

Characteristic	Little/no attendance + online use (n=28)			Little/no attendance or online use (n=110)		
	RR^a^	*P*	95% CI	RR	*P*	95% CI
Deployed	0.45	.069	0.19-1.07	0.57	.049	0.32-1.00
Education	1.40	.107	0.93-2.12	1.11	.424	0.85-1.46
Employed	0.55	.274	0.19-1.60	3.10	.012	1.29-7.47
Child age	1.18	.064	0.99-1.41	1.21	.005	1.06-1.38
Incentives	15.94	.010	1.96-129.69	1.70	.184	0.78-3.70

^a^RR denotes relative risk. Reference group attended at least 4 times but had little to no online use (n=77).

The second hypothesis that military-related variables would be associated with patterns of lesser online and face-to-face use was partially supported. To avoid the confound between gender and deployment, only those men (n=173) and women (n=34) who had been deployed were selected in the final model and gender was added into the model (n=207). The final model fit the data well (χ^2^=40.67_24_, *P*=.004). Compared with parents who attended primarily face-to-face, mothers who had been deployed were nearly 4 times more likely to have moderate online use and attend face-to-face and were more than 4 times more likely to attend primarily online, although, likely because of the relatively small number of women, the effect is only marginally significant (see Tables 5 and 6). Compared with parents who attended primarily online, those with fewer months deployment were less likely to have a pattern of high online use and face-to-face attendance.

**Table 5:** Multinomial logistic regression of characteristics associated with patterns of online component use and face-to-face attendance among those who had been deployed (n=207).

Characteristic	Attendance + high online use (n=34)			Attendance + moderate online use (n=43)		
	RR^a^	*P*	95% CI	RR	*P*	95% CI
Months deployed	0.78	.041	0.61-0.99	0.88	.265	0.71-1.10
Education	1.53	.029	1.04-2.24	1.31	.140	0.91-1.89
Employed	0.47	.334	0.10-2.18	0.77	.718	0.18-3.24
Child age	1.05	.617	0.87-1.26	1.01	.919	0.85-1.20
Female	1.15	.849	0.28-4.71	3.78	.023	1.21-11.83
Incentives	11.20	.004	2.14-58.60	2.80	.068	0.92-8.49

^a^ RR denotes relative risk. Reference group attended at least 4 times but had little to no online use (n=55).

**Table 6:** Multinomial logistic regression of characteristics associated with patterns of online component use and little face-to-face attendance among those who had been deployed (n=207).

Characteristic	Little/no attendance + online use (n=13)			Little/no attendance or online use (n=66)		
	RR^a^	*P*	95% CI	RR	*P*	95% CI
Months deployed	0.92	.563	0.69-1.23	0.86	.164	0.69-1.06
Education	1.25	.402	0.74-2.09	1.16	.389	0.83-1.62
Employed	0.50	.503	0.64-3.86	0.69	.567	0.19-2.46
Child age	1.12	.394	0.87-1.43	1.16	.044	1.00-1.35
Gender	4.41	.095	0.77-25.20	1.00	.994	0.29-3.50
Incentives	8.69	.044	1.06-71.22	2.03	.113	0.84-4.92

^a^RR denotes relative risk. Reference group attended at least 4 times but had little to no online use (n=55).

## Discussion

To better understand patterns of engagement in a preventive parenting intervention program with both online components and face-to-face sessions, we sought to identify demographic, programmatic, and ecological characteristics of military life related to parents’ use of online components and face-to-face attendance. As in prior studies evaluating PMTO (personal oral communication, Marion Forgatch, PhD, January, 2014), most participants had a high frequency of attendance (on average 10/14 sessions) once they participated in more than 3 face-to-face sessions. We also found this to apply to online components, such that parents who completed more than 3 online components had a high frequency of online component use (on average, 41/52 components). The high percentage of total participation (84.5% of families, 78.9% of individuals) was comparable to or better than other parenting programs [32,37]. Five patterns of engagement were identified: high online use with face-to-face attendance, moderate online use with face-to-face attendance, primarily face-to-face attendance, primarily online use, and little to no online use or attendance.

This study found that among those who had been deployed, participants with a high number of months of deployment were less likely to be in the group with the highest engagement both online and face-to-face. Although military families communicate via Internet-based medium during deployment [52,53], it may be that at home those who spent the most time away value face-to-face time with family rather than spending time on the computer. Alternatively, these individuals may be less connected to parenting, given their lengthy deployments and absences from the family, and thus see less value in participating in a parenting program. Employment was not significantly associated with participation among those who had been deployed, but it was significantly associated with lower participation across the entire sample, which suggests that employment of the nondeployed spouse may have been a barrier to participation.

Incentives seemed to be important motivators for parents to participate in online components; these findings parallel past findings regarding the use of incentives with face-to-face attendance [43,45]. Parents participating before the introduction of incentives (ie, phase 1) had low participation online, but an increase in online participation was evident in phases of delivery where incentives were offered to participate online. Although the proportion of those who participated online among those who were incentivized with a drawing in phase 2 was similar to those who were incentivized directly with a gift card in phase 3, it should be noted that those who were recruited in phase 3 were “hard to reach” participants who had not previously been able to attend face-to-face. Almost 10% of families who participated in ADAPT did so primarily online and likely would not have participated in the program at all without the online option. Overall, this evidence suggests that online options (especially those that are incentivized) could increase engagement in parenting programs and provide access to resources for parents who are isolated or unable to attend face-to-face programs.

Similar to previous research findings [27], mothers were more likely to engage with online parenting content than fathers. This study is the first to demonstrate that mothers who had been deployed were more likely to participate in an online setting compared with participation in the traditional face-to-face setting. Deployed mothers may be a particularly appropriate target population for online programs, given these findings, and their likely high use of online technologies during deployment.

We also found that those with the highest levels of education were the most engaged online. This finding corroborates past research that found that parents with higher income levels were more likely to prefer online services and seek parenting information online [21,24] than low-income parents. We also found that parents with a 4-year degree were significantly more likely to access knowledge checks (quizzes) and handouts but not video components or mindfulness exercises, compared with those without a 4-year degree. Although parents in lower socioeconomic circumstances increasingly have access to online content, past research has suggested that digital literacy deficits may continue for some individuals because they lack skills or time to process online content [22]. For example, less educated parents may not feel that they have the confidence to complete quizzes. These findings suggest that demographic characteristics are important to consider in engaging military families in online and face-to-face parenting programs.

### Limitations and Future Directions

Although this study is novel in its examination of demographic, programmatic, and ecological characteristics of military life as they relate to participation in an Internet-enhanced parenting program, there are some limitations to consider. This study included a sample of Midwestern NGR families with a parent who had been deployed, the majority of whom were European American. Although our findings help us better understand program engagement in this important subset of parents, our results may not generalize to active duty military families or civilian families. In addition, past research has found barriers to participation among those with low SES, but this sample did not have high variability of SES among participants (ie, most were upper middle class), and therefore the results may not adequately reflect the socioeconomic challenges to participating in a parenting program online. We found interesting differences in online component use based on cohort and incentives to participate online, but we did not randomize families to receive incentives (or not) at the beginning of the study. Incentives were added only in 2 later phases of the study. Randomized controlled trials examining the influence of incentives to participate in online supplements would provide important, additional evidence regarding their influence on participation. Additionally, in this study, the number of clicks was used as a proxy for completion of online modules; however, better measures of the time participants spend online and quality of those interactions are needed.

This study provides a foundation for future research on engaging parents in various contexts. While important differences were found when analyzed by parents’ demographic characteristics, program incentives, and the ecological context, future research that includes parents’ psychological characteristics, such as motivation for participating in parenting programs, self-efficacy as a parent, and other unique individual characteristics, may further elucidate differences in participation. Additionally, research is needed to understand how engagement with online components versus face-to-face participation translates into behavior change (eg, improved parenting and child adjustment). Dismantling trials (which disaggregate different online components and face-to-face interventions options) as well as randomized preference trials (which randomize participants to choose or be assigned their modalities of choice) would provide practitioners and researchers with important information about what works in engaging and serving families through preventive parenting programs.

### Conclusions

This research underscores the need to understand patterns of parent engagement in both Internet-based and face-to-face parenting programs. Our findings add to existing evidence that Internet-based participation has the potential to increase some parents’ participation; however, face-to-face programming also appears to provide unique benefits to some parents, which could include instrumental and emotional support. Collecting data and monitoring the use of online components and face-to-face participation in parenting programs provides opportunities to better understand audiences and potentially improve both engagement and retention [7].

## Appendix

Multimedia Appendix 1 Examples of web pages from ADAPT’s online supplement.

## Abbreviations

ADAPT: After Deployment, Adaptive Parenting Tools
NGR: National Guard and Reserves
PMTO: Parent Management Training–Oregon Model
RCT: randomized controlled trial
SES: socioeconomic status
