# Supplementary figures and images for: If You Build It, Will They Come? Patterns of Internet-Based and Face-To-Face Participation in a Parenting Program for Military Families
Source: J Med Internet Res. 2016 Jun 22;18(6):e169. doi: 10.2196/jmir.4445 (PMC4935796; doi:10.2196/jmir.4445)

## Slide 1
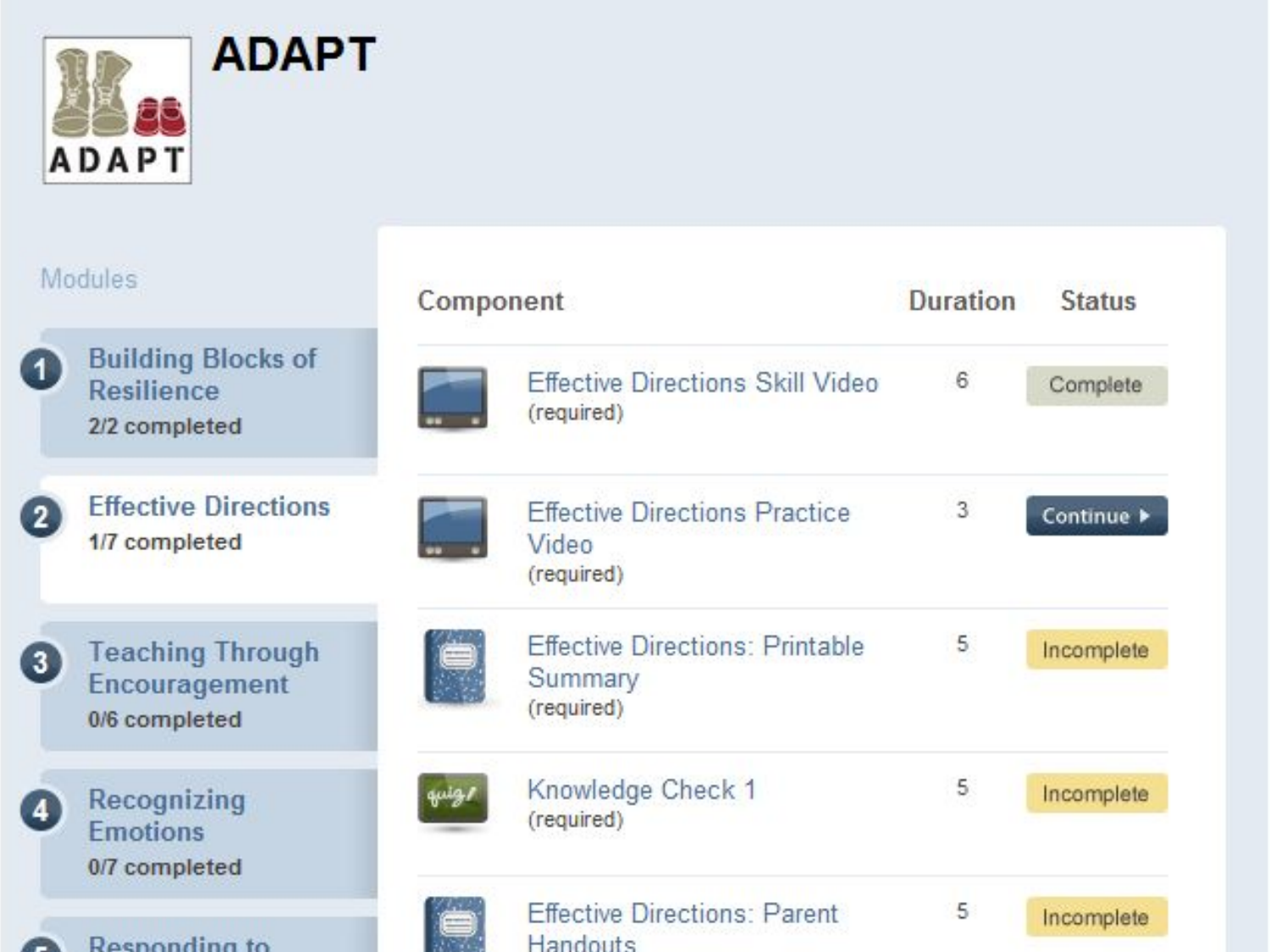

## Slide 2
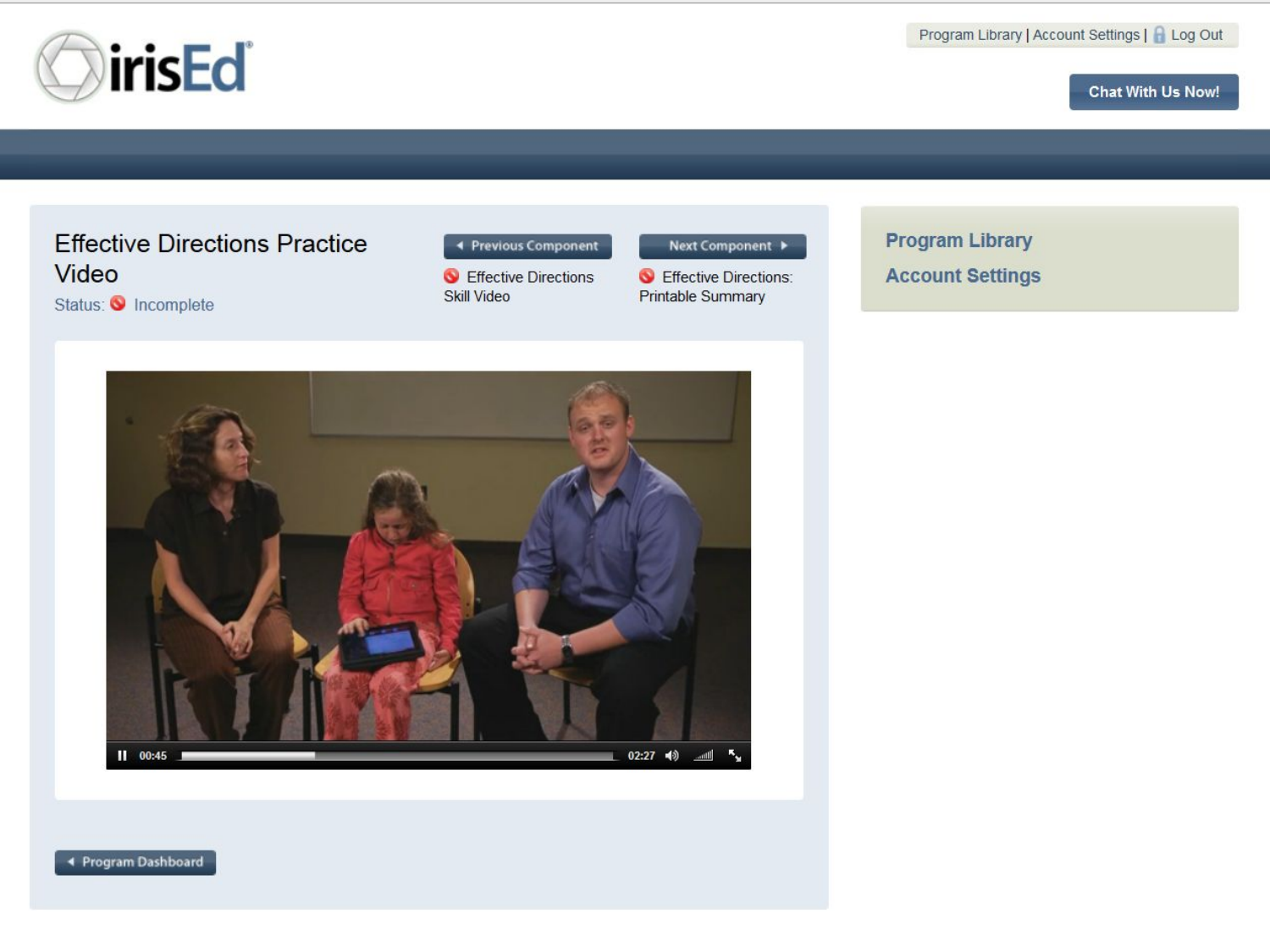

## Slide 3
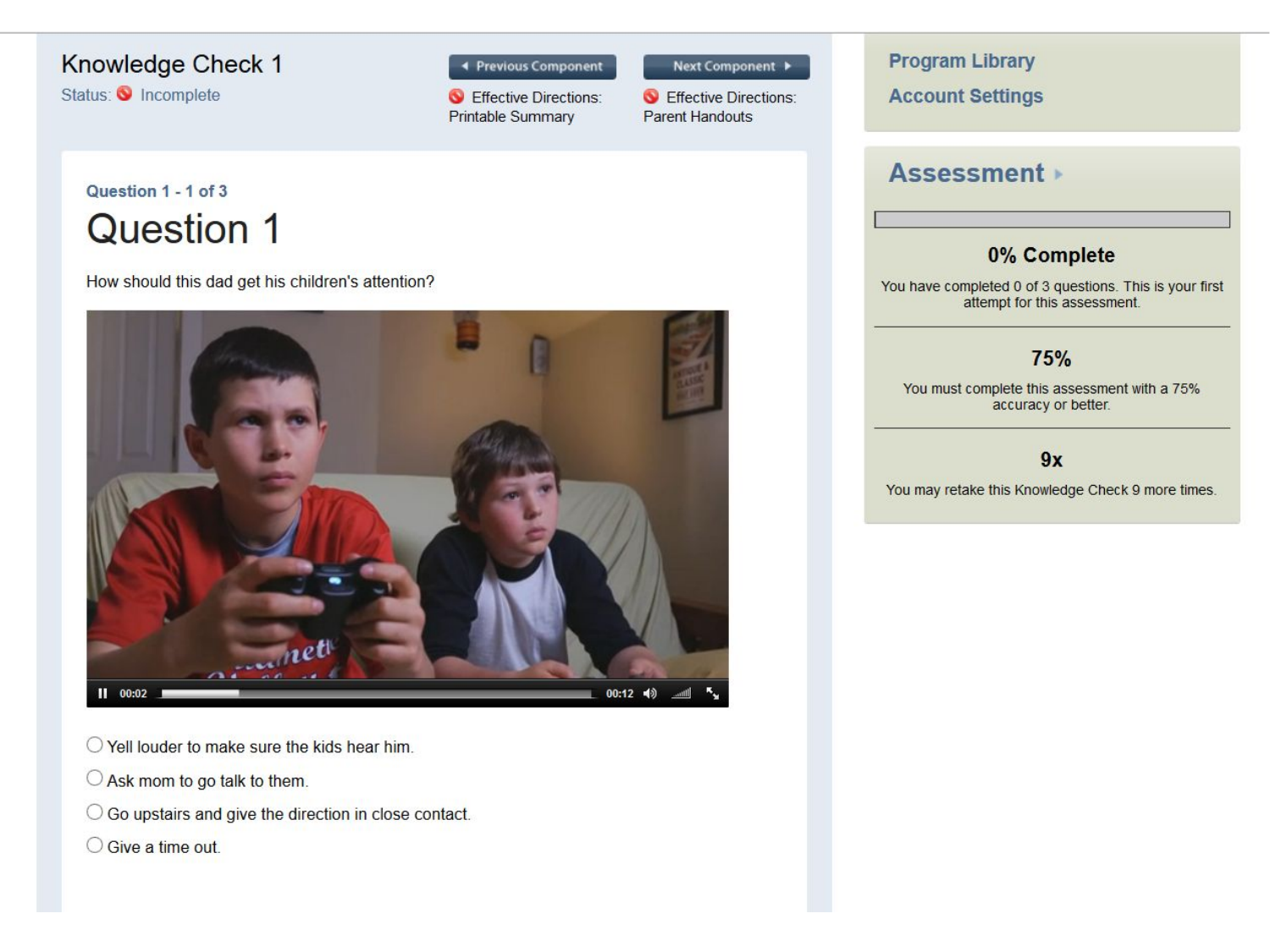

Supplement: Multimedia Appendix 1 [file jmir_v18i6e169_app1.pptx]
